# Associations between emotions expressed in internet news and subsequent emotional content on twitter

**DOI:** 10.1016/j.heliyon.2022.e12133

**Published:** 2022-12-05

**Authors:** Eric Mayor, Marcel Miché, Roselind Lieb

**Affiliations:** University of Basel, Switzerland

**Keywords:** Emotions, Social media, News media, LIWC, GDELT

## Abstract

We report on the first investigation of large-scale temporal associations between emotions expressed in online news media and those expressed on social media (Twitter). This issue has received little attention in previous research, although the study of emotions expressed on social media has bloomed owing to its importance in the study of mental health at the population level. Relying on automatically emotion-coded data from almost 1 million online news articles on disease and the coronavirus and more than 6 million tweets, we examined such associations. We found that prior changes in generic emotional categories (positive and negative emotions) in the news on the topic of disease were associated with lagged changes in these categories in tweets. Discrete negative emotions did not robustly feature this pattern. Emotional categories coded in online news stories on the coronavirus generally featured weaker and more disparate lagged associations with emotional categories coded in subsequent tweets.

## 1 Introduction

Many individuals follow health-related news (Brodie et al., 2003), and the SARS-CoV-2 novel coronavirus disease discovered in 2019 (COVID-19) has been a topic of continuing broad interest and coverage, notably due to its spread and potentially fatal outcome. We hence postulated that a large majority of Twitter users were regularly exposed to these topics directly through the news or on social media through news-sharing posts of other users (Dafonte-Gómez, 2018; Kalsnes and Larsson, 2018). Drawing on research on emotion contagion in interpersonal (e.g., one to one with direct contact generally) and social media (e.g., one to many, without direct contact generally) settings, we further hypothesized that Twitter users were emotionally affected by the emotional content of the news on these topics. Indeed, emotions in news stories can evoke similar emotions in readers. For instance, studies have shown that the negative framing of news has a corresponding effect on readers’ emotions (e.g., Lecheler et al., 2013; Hawton et al., 2021). Importantly, negative emotions increase following crisis-related news (Garfin et al., 2020). However, research so far has not investigated the relation between emotions expressed in the news media and those subsequently expressed on social media.

Here, we report on the first investigation of large-scale temporal associations between emotions expressed in online news media and emotions subsequently expressed on social media (Twitter). We investigated the lagged associations between emotions coded in internet news articles featuring the topics of disease and the coronavirus and tweets generally, from January to September 2020. We expected that daily aggregated changes in emotions in news articles would be associated with corresponding changes in subsequent tweets.

## 2 Emotions in tweets

Emotions are frequently expressed in posts on social media platforms (e.g., Bazarova et al., 2015). Emotions expressed in tweets have been shown to have value in distinguishing favourable and unfavourable audiences of popular accounts on Twitter (Bae and Lee, 2012), in predicting election results (Singh et al., 2020), and in differentiating between atheists and believers among users (Ritter et al., 2014). Data from Twitter and other social media platforms have also successfully been used to detect depression (Guntuku et al., 2017; Kim et al., 2021). Indeed, emotion-coded Twitter data collected on a large scale can be considered a ‘barometer’ of the wellbeing of a population (Golder and Macy, 2011; Mayor and Bietti, 2021; Mayor et al., 2022; Massell, Lieb, Meyer & Mayor, in press). A recent topic of interest has been the detection of real-time changes in emotions expressed on Twitter (Tasoulis et al., 2018). Finally, a small number of studies have examined emotional contagion on social media (e.g., Ferrara and Yang, 2015; Kramer et al., 2014). The study of emotions in tweets has expanded in recent years. The field has focused mostly on changes in emotions following catastrophic events (e.g., El Bacha and Zin, 2018; Khun et al., 2019; Jones and Silver, 2020; Wang and Taylor, 2018; Yum, 2020). For example, a study (Jones et al., 2016) using tweets coded for emotional content with the Linguistic Inquiry and Word Count (LIWC) tool (Pennebaker et al., 2015) found that negative emotions among campus account followers increased after college campus violence. Similar patterns were observed during the first COVID-19 lockdown period (e.g., Machuca et al., 2021) and, more generally, at the onset of the pandemic (Ashokkumar and Pennebaker, 2021). In other words, emotions expressed on Twitter become more negative during crises, but to date, this finding has not been related to the emotional content of actual news.

## 3 Emotions in the news

The role played by emotions in the news has frequently been investigated in experiments in relation to policy. Different emotions in the news have often been associated with different readers’ policy preferences and decisions (e.g., Nabi, 2003). The impact of manipulated emotions in the news on policy preferences was found to be mediated by emotions elicited in the reader (Kühne and Schemer, 2015). The emotional framing of news can be used to polarize the public on targeted issues (Clifford, 2019) and to induce differences in the processing of subsequent news (Baumgartner and Wirth, 2012). The role of different emotions in opinion formation depends upon the issue or policy at hand (Druckman and McDermott, 2008); that is, negative emotional appeals are not robustly linked with the adoption of the intended opinion or behaviour. For instance, eliciting hope rather than fear is related to support for policies that target climate change (Moser, 2007; Nabi et al., 2018).

According to Ungerer (1997), emotions are encoded by journalists in the news and decoded from this medium by readers, who are correspondingly being induced to feel a certain way. This process might be pragmatically exploited by journalists to increase their readership through news-sharing and through readers’ comments on their articles (Orgeret, 2020) and to improve attention to and recall of their pieces (e.g., in audiovisual news: Lang, 2000; Lang et al., 1996). Complementarily, McIntyre and Gyldensted (2018) propose that journalists voluntarily focus on including constructive coverage by notably incorporating positive emotions and solutions to the issues raised in their texts. Adding a silver-lining to an otherwise negative story can also alleviate the increase in negative emotions felt after consuming otherwise negative news (McIntyre and Gibson, 2016). Following this line of reasoning, research has found that readers of news deliberately written to be constructive had higher positive emotions than readers of more traditional news (Hermans and Prins, 2022).

Only a few studies have quantitatively examined emotions shared in actual news content. For instance, using affect-coded news data from the Global Database of Events, Language, and Tone (GDELT; Leetaru and Schrodt, 2013), a recent study showed that affect in the news predicted macro-economic indicators (Tilly et al., 2021). Ebola-related sentiment follows similar trajectories in the news and in social media (Kim, Jeong, Kim et al., 2015). Another study found that news related to the COVID-19 pandemic featured geo-temporal variations in depressiveness (Alambo et al., 2020). A study analysing expressed emotions in COVID-19-related news headlines established that negative emotions (e.g., fear, sadness, anger) were prominent in news headlines, the negativity of which strongly increased over time (Aslam et al., 2020). The authors propose that emotions shared in the news affect the emotions felt by readers. The literature presented at the beginning of this section supports this assumption.

## 4 The current study

### 4.1 News exposure

Twitter users regularly share news media content through their posts (Kalsnes and Larsson, 2018), and internet users’ search interests seem to align with internet news media coverage (Kwak et al., 2018). In particular, individuals closely follow news related to health (4 in 10 individuals; Brodie et al., 2003), and the coronavirus has been, and remains, a topic of broad interest and coverage. Indeed, when death looms as the possible outcome of an infection, most individuals seem to pay close attention. For instance, Tsao et al. (2021) reviewed many studies on social media use which indicate that once human-to-human infection of COVID-19 was officially confirmed in China, public attention increased quickly and remained stable over time. It is hence reasonable to postulate that a large majority of Twitter users are regularly exposed to these topics through the news or on social media through news-sharing posts of other users, particularly if the news has a high emotional polarity (Kalsnes and Larsson, 2018; Khuntia et al., 2016).

### 4.2 Emotion contagion

The propagation of emotions among individuals has been a subject of research for decades in a diversity of settings such as informal interaction (Cacioppo et al., 2009; Fowler and Christakis, 2008; Kimura et al., 2008), psychotherapy (e.g., Hsee et al., 1992) and organizational life (for a review, see Barsade et al., 2018). The emotions that have been found to be propagated range from happiness to loneliness (Cacioppo et al., 2009) and depression (Rosenquist et al., 2011). Emotions are propagated in the short and long term (Hatfield et al., 2014; Barsade et al., 2018). In face-to-face interaction, research has highlighted the relationship between facial mimicry and emotion contagion in that “people tend to feel emotions consistent with the facial expressions they adopt” (Hatfield et al., 2014, p.162). Yet, research has demonstrated that facial mimicry is not necessary for emotion contagion, as it can occur without facial cues (e.g., Dezecache et al., 2013). In decentralized work groups interacting using a chat, Hancock et al. (2008) found that participants interacting with individuals induced to feel sad use less positive affect words compared with participants interacting with individuals who went through neutral emotion induction. In another similar study, Guillory et al. (2011) found that participants interacting with individuals induced to feel anger notably reported feeling more tense, although they could not see or hear their partners in interaction. These findings show that emotion contagion occurs in indirect interaction without face-to-face or verbal contact. Therefore, neither non-verbal cues nor direct interaction are necessary for emotional contagion to occur. Social media research has shown that expressed emotions are propagated among social media users (e.g., Coviello et al., 2014; Kramer et al., 2014).

Emotions in news stories, too, may evoke similar emotions in readers (Ungerer, 1997). Such associations are suggested by studies showing that the negative framing of news has an effect on emotions felt by readers (e.g., Kühne and Schemer, 2015; Lecheler et al., 2013). One prominent example based on such research findings is the World Health Organisation's guidelines on media coverage of suicide (World Health Organization, 2017), the importance of which is being emphasized especially in relation to the COVID-19 pandemic (Hawton et al., 2021). A princeps study (Veitch and Griffitt, 1976) found that good news elicited positive affect, whereas bad news elicited negative affect. Balzarotti and Ciceri, 2014 reported that the emotional content of manipulated news was congruently related to readers' emotions. Similarly, de Hoog and Verboon (2020) report that the emotions felt following news consumption are concordant with the perception of the negativity of the news, an association which was moderated by perceived relevance of the news. Other studies found that the emotional valence of the news congruently affects participants' emotional states: Johnston and Davey (1997) showed that negatively valenced news increased participants' sadness and anxiety. McIntyre and Gibson (2016) discovered differences in positive affect between all comparisons of positively, negatively, and neutral-valenced stories, with positive stories eliciting the most positive affect. Focusing on negative news only, Szabo and Hopkinson (2007) found that exposure to such news negatively affected the mood of participants, an effect that could be moderated by relaxation techniques. Furthermore, as noted above, a study by Hermans and Prins (2022) has shown that in comparison to those who read traditional news, readers of news deliberately written to be constructive (inclusion of positive emotion words, mentions of solutions to issues) reported higher levels of positive emotions.

Additionally, crisis-related news coverage can have a negative impact on physical and mental wellbeing (Garfin et al., 2020) which is related to experienced emotions (Gloria and Steinhardt, 2016; Gross and Muñoz, 1995). The literature reviewed above suggests that negative and positive emotional news content elicits concordant emotions in news consumers. Yet the need for a longitudinal study of the relation between emotions in the news media and on social media has so far been overlooked. The main goal of this study was to examine the temporal associations of emotions in the news and in tweets on a large scale, using internet news articles as a proxy for the overall news climate.

We hence investigated the temporal associations between emotions coded in internet news articles on disease and the coronavirus and tweets generally over a period of 9 months (from January to September 2020). We expected that emotions in tweets would closely follow emotions expressed in the news pages.

## 5 Method

### 5.1 Samples

All data used in this study are publicly available.

#### 5.1.1 News articles

We collected coded data from online news articles using the Global Knowledge Graph (GKG) of the GDELT (Leetaru and Schrodt, 2013). “The GDELT Project monitors the world's broadcast, print, and web news from nearly every corner of every country” (https://www.gdeltproject.org). The GKG notably provides freely available content-coded data of online news and other sources over a large time span, totalling several hundreds of millions of entries. We selected articles related to the topics of ‘disease’ and ‘coronavirus’. All coronavirus articles were also part of the topic of ‘disease’ but were treated separately. The beginning of the period of interest for this study was January 2020. Because of large gaps in the GKG data for October and November 2020, the end of the period of interest for this study was September 30, 2020. We used data from online news articles from the following sources: reuters.com, msn.com, yahoo.com, forbes.com, cnbc.com, nbcnews.com, foxnews.com, usatoday.com, washingtonpost.com, washingtontimes.com, nytimes.com, latimes.com, thehill.com, chron.com, inquirer.net, and thetelegraph.com. These sources were selected according to the criteria of location (USA) and frequency of online news articles published (more than 10,000 articles from each source in the corpus) and on the basis of our subjective assessment of their reputation. The total number of coded articles in the sample approximated 1 million (*N* = 222,674 for the topic of disease and *N* = 772,702 for the topic of coronavirus). These sample sizes correspond to all matching entries for the mentioned period and sources. We believe such large sample sizes are appropriate because of the duration of the period of interest.

#### 5.1.2 Tweets

For the collection of Twitter data, we first selected 50,000 users (located in the 160 most populated U.S. counties; convenience sample size) using stratified sampling from a pre-existing dataset (Mayor and Bietti, 2021). We then requested the download of each of these users' timelines (up to 3,200 tweets per user) using the *rtweet* package in R. The timelines of 39,196 users were retrieved (reasons for non-retrieval: account deleted or suspended, privacy settings). We excluded users who did not publish any tweets in January 2020. From those, we also excluded users who did not publish any tweets after January 2020 during the period of interest, so that the data reflected change in users’ emotions after that month as well (25,220 users remained). The total number of tweets in the sample was 6,218,192 (per user: *M* = 246.56, *SD* = 317.19). The duration of the research period calls for a sample size of this magnitude.

#### 5.1.3 Data coding and preparation

##### 5.1.3.1 News articles

Coding of news article data relevant to this study is already available from the GKG: We selected categories of the LIWC (Pennebaker et al., 2015) relating to positive emotions, negative emotions, anger, sadness, and anxiety. We group-centred each variable by source and averaged the data by day. The resulting variables therefore expressed aggregated change from source averages.

##### 5.1.3.2 Tweets

After preprocessing (removal of user mentions, hashtags, links, and emojis as well as carriage returns and leading, trailing, and repeated whitespace characters), we coded the tweets using LIWC 2015. We selected the same variables as for the news articles. Each variable was group-centred by user. The resulting variables, therefore, expressed change from users’ average scores. These variables were then aggregated in two steps: First, the data were aggregated by date and user (so that each user with tweets on a given day contributed equally to the second aggregation step). Second, the resulting data were then averaged by day.

### 5.2 Data analysis

We relied on variable-by-variable cross-correlations using R to analyse the data aggregated after group-centring (lags of -4 days to +4 days). The cross-correlations between two variables representing time series, for instance A and B, are the correlations between the variables with a range of lags between the two. Cross-correlations, therefore, allow estimating the associations between time series at various delays (for instance A preceding B by one time unit, so a lag of -1; or B preceding A by one time unit, so a lag of +1), not simply when they are synchronized as in the case of the zero-order correlations. If the variables are centered (as is the case in this study), a significant correlation of .2 at lag -1 between variables A and B can be interpreted as, when A leads B by one time unit, change in variable A is positively associated with change in variable B (or change in variable A precedes change in variable B at lag -1).

More specifically, each selected LIWC aggregated variable in the aggregated news article data was cross-correlated with its counterpart in the aggregated Twitter data. As there were no news articles on the topic of coronavirus in the selected sources for January 1, 2, and 5, we analysed the data beginning with January 6, 2020. The end of the period of interest was September 30, 2020. Because of moderate to strong autocorrelations in the time series of several emotional categories, we analysed the data with and without correction for autocorrelation using an approach similar to the two-step approach (see Bence, 1995), that is, using the residuals of models regressing each variable at lag 0 on their values at lag -1.

## 6 Results

### 6.1 Positive emotions

**Figure 1:**
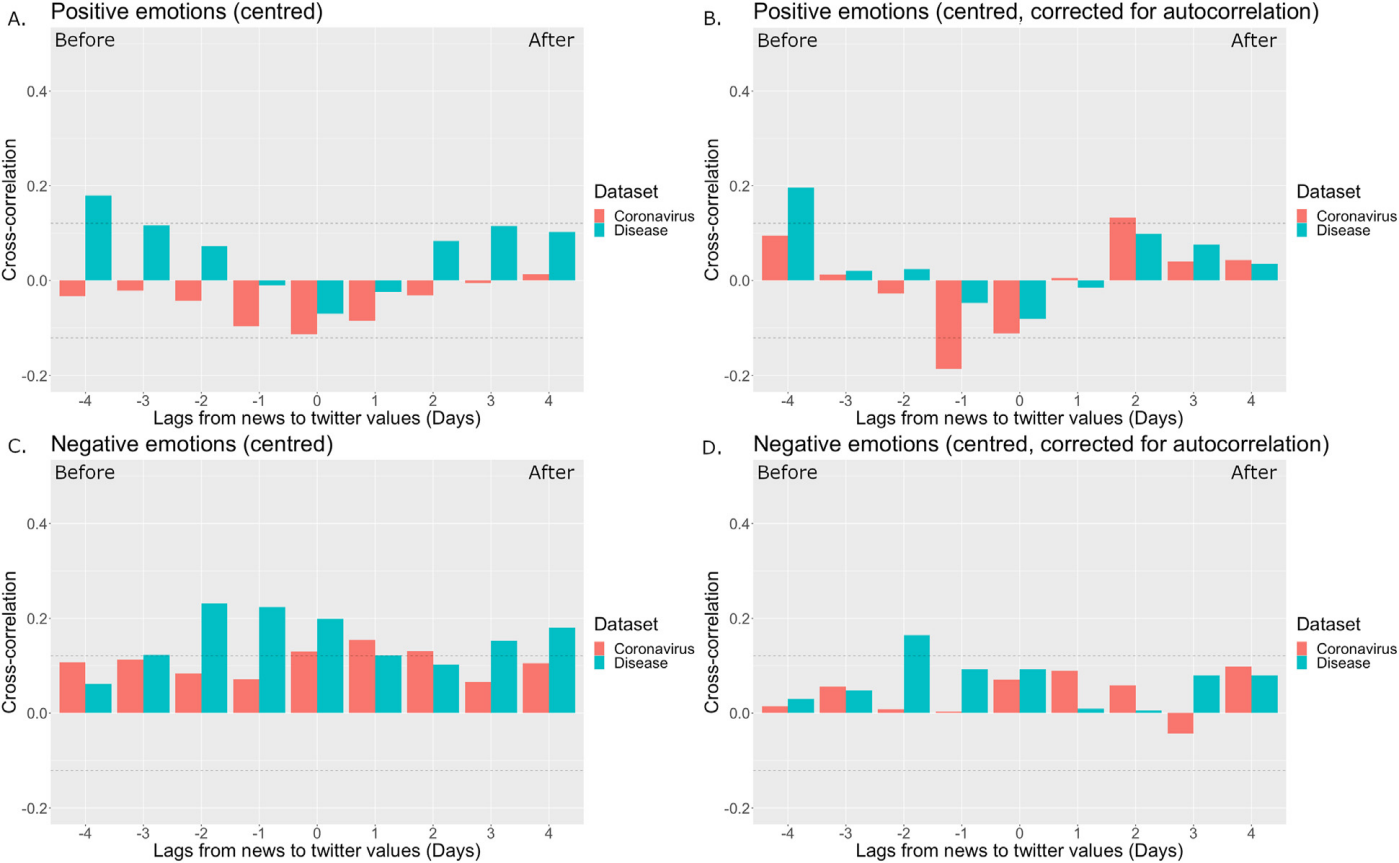
Cross-correlations of positive emotions (Panels A, B) and negative emotions (Panels C, D) in the news on topics Coronavirus (Orange) and Disease (Green) with these emotions in tweets. Dashed lines indicate significance at *p* < .05.

The results for positive emotions are presented in the top panels of Figure 1 (and in Supplementary table 1 with confidence intervals and exact *p* values). Positive emotions in tweets were associated with positive emotions in the news on disease 4 days earlier with and without correcting for autocorrelation in positive emotions (Panels A and B). No significant cross-correlations were observed between positive emotions in tweets and in the news on the coronavirus when not correcting for autocorrelation (Panel A). When correcting for autocorrelation (Panel B), positive emotions in tweets were negatively associated with positive emotions in the news on this topic 1 day earlier, and positively associated with positive emotions in such news 2 days after.

#### 6.1.1 Negative emotions

The results for negative emotions are presented in the bottom panels of Figure 1. Negative emotions in the tweets were associated with negative emotions in articles on disease on the same day as well as 1 and 2 days before and 3 and 4 days after when not correcting for autocorrelation (Panel C), and with negative emotions in such news 2 days before when correcting for autocorrelation (Panel D). Negative emotions in tweets were associated with negative emotions in the news relating to the coronavirus on the same day as well as 1 and 2 days after when not correcting for autocorrelation (Panel C), but not when correcting for autocorrelation (Panel D).

#### 6.1.2 Anxiety

**Figure 2:**
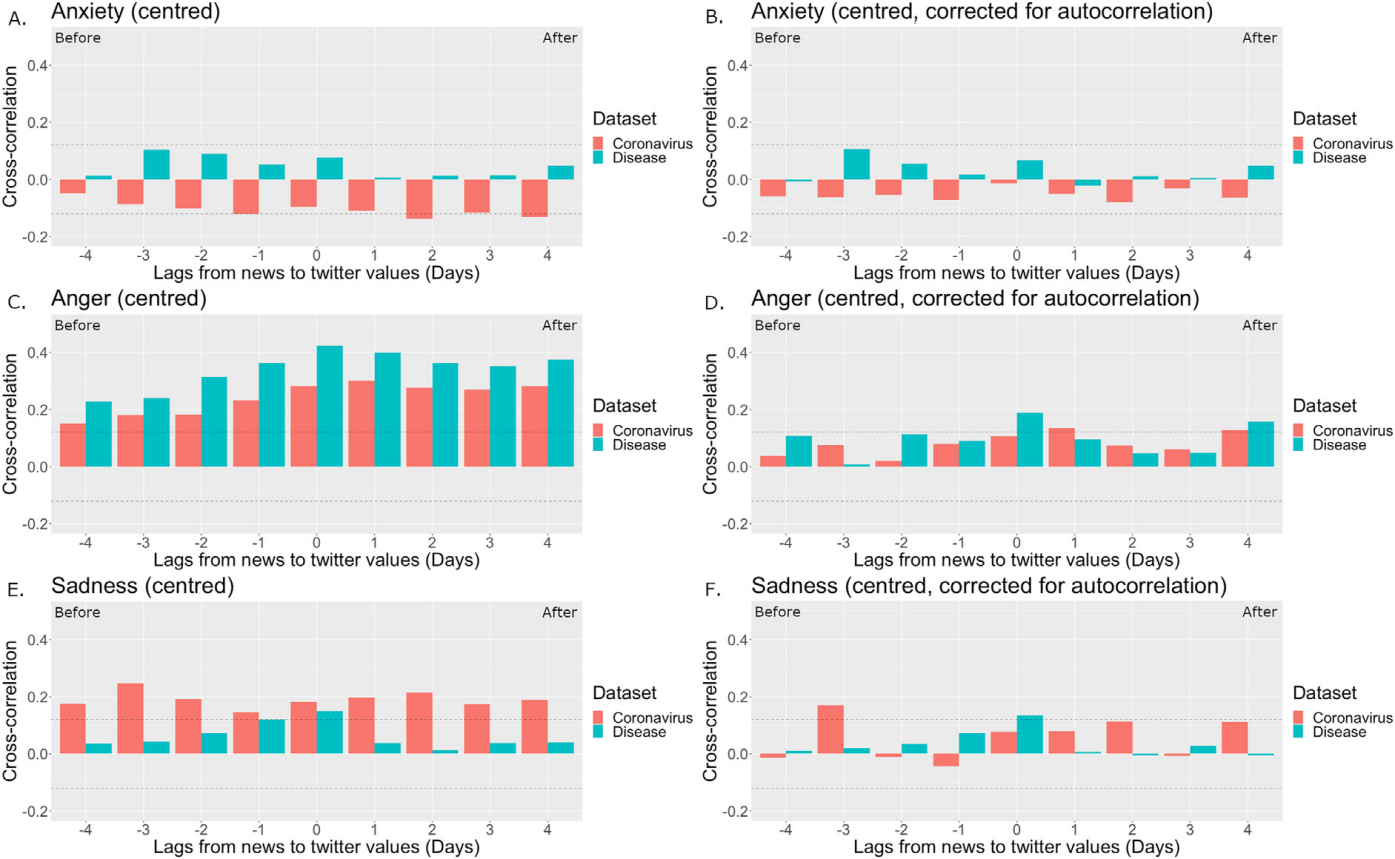
Cross-correlations of anxiety (Panels A, B), anger (Panels C, D), and sadness (Panels E, F) in the news on topics Coronavirus (Orange) and Disease (Green) with these emotions in tweets. Dashed lines indicate significance at *p* < .05.

The results for anxiety are presented in the top panels of Figure 2. No significant associations were found between anxiety in the tweets and anxiety in the news on disease*.* Anxiety in the tweets were negatively associated with anxiety in the news relating to the coronavirus 2 and 4 days later when not correcting for autocorrelation (Panel A), but not when correcting for autocorrelation (Panel B).

#### 6.1.3 Anger

The results for anger are presented in the middle panels of Figure 2. Anger in tweets was positively associated with anger in the news on both topics from 4 days before to 4 days after when not correcting for autocorrelation. When correcting for autocorrelation, positive associations were observed between anger in tweets and anger in the news relating to disease on the same day and 4 days after. In autocorrelation-corrected data, anger in tweets was associated with anger in the news on the coronavirus 1 day later and 4 days later.

#### 6.1.4 Sadness

The results for sadness are presented in the bottom panels of Figure 2. Sadness in tweets was positively associated with sadness in the news relating to disease on the same day with or without correction for autocorrelation. Positive associations were found between sadness in tweets and sadness in the news on the coronavirus from 4 days before to 4 days after when not correcting for autocorrelation (Panel E). When correcting for autocorrelation (Panel F), sadness in the tweets was positively associated with sadness in the news on this topic only 3 days before.

## 7 Discussion

Emotions expressed by many people on social media can be considered an indicator of the psychological wellbeing of the population, and past research has notably shown the existence of 24-hr and 7-day variations (Mayor and Bietti, 2021) that can be found across cultures (Golder and Macy, 2011). The impact of catastrophic events on emotions in collectivities has also been highlighted (e.g., Jones et al., 2016). We conducted the first investigation of large-scale temporal associations between emotions expressed in online news media and emotions subsequently expressed on social media (Twitter). As expected, we found temporal (lagged) associations between changes in positive and negative emotions coded in online news on the topic of disease and subsequent changes in these emotions in tweets: Changes in these emotional categories in the news were typically associated with corresponding changes in the tweets (in closer proximity for negative emotions: see Figure 1), both in time series corrected for autocorrelation and in time series without such correction.

This pattern was not robustly found with regard to discrete negative emotions (see Figure 2), which might be explained by the interrelatedness of discrete emotions (Pe and Kuppens, 2012). Similarly valenced emotions can increase together (augmentation), whereas emotions of opposite valence tend to grow or decline in opposite ways (blunting). However, previous research has also shown that discrete emotions can follow different trajectories during tragedies (e.g., Doré et al., 2015). In the current study, no significant temporal associations were found between changes in anxiety in the news on the topic of disease and in tweets. Only same-day associations were found between disease-related news and tweets regarding changes in sadness. The pattern of results was strikingly different between autocorrelation-corrected data and non-corrected data with regard to changes in anger. Overall, the pattern of results suggests a rapid (same-day) correspondence of changes in discrete negative emotions (sadness and anger) between disease-related news and tweets, and a more delayed correspondence for generic emotional categories.

Findings regarding associations of changes in emotional categories in coronavirus-related news and in tweets are more disparate. We observed a negative association between changes in positive emotions in online news and in tweets the day after in data corrected for autocorrelation only, which was contrary to what we expected. In non-corrected data, changes in negative emotions in the news were associated positively with subsequent changes in such emotions in tweets on the same day, as found for the topic of disease. Changes in discrete negative emotions in the news did not feature robust lagged associations with next-day changes in tweets except for lagged associations of changes in sadness (3 days before in the news).

Overall, the general pattern of results shows that changes in emotional categories coded in online news about the coronavirus generally featured weaker lagged associations with subsequent changes in emotional categories coded in the tweets compared with those found with the news on the topic of disease. One explanation for these differentiated results could be that other sources of information played a more important role than journalistic online news on the coronavirus, compared with news stories on disease more generally. For instance, the coronavirus was a topic with broad public engagement on social media which featured correct and incorrect information (e.g., Kouzy et al., 2020; van Der Linden et al., 2020). In some instances, we found data in the tweets to be associated with data in the news days later—a finding that we did not expect.

Overall, our study suggests that positive and negative emotions in internet news stories on diseases are globally associated with the subsequent emotional experience of individuals. Drawing on appraisal models of emotions, experimental news media studies have shown that different elicited emotions are related to different preferences for policies (e.g., Nabi, 2002, 2003). Emotions have also been shown to be moderators of how subsequent news are perceived (Druckmann and McDermott, 2008). Although we did not address such questions in this study, it is nevertheless probable that news-media-induced changes in experienced emotions can be consequential in shaping the opinions of individuals, the way individuals process forthcoming new information, and their behaviour in daily life. Further, an accumulating body of evidence shows that emotions play a crucial role in shaping social relationships (e.g., Van Kleef, 2009).

The main strength of our study is its exceptional ecological validity in the field, and its main weakness is the lack of control of potentially confounding variables. Further, we only included tweets and news sources from the U.S. Hence, the generalizability of our results is limited. Our study did not distinguish between individual and organizational accounts as this is not a variable that is provided by Twitter. Another limitation is the study's use of aggregated group-centred data from millions of Twitter users and hundreds of thousands of articles, and hence the impact of emotions in specific articles on individual measurements could not be estimated due to the fact that no links exist between individual Twitter users and the specific news articles they may have read. Finally, since with our approach it was only possible to study associations and not causation, we cannot ascertain if the news caused the emotions expressed in Twitter posts.

## 8 Conclusion

This study offers a unique contribution by focusing on the associations between emotions expressed in the news media and emotions expressed on social media. We have shown that generic emotions (unlike discrete emotions) in news on the topic of disease typically precede corresponding emotions in tweets. This possibly corresponds to a framing effect of the news media on emotions as tested in experimental studies (Lecheler et al., 2013) and frequently observed in the formation of personal opinions (e.g., Scheufele, 1999). Such effect might be investigated in more depth in further studies.

## Declarations

### Availability of data and materials

The dataset (aggregate scores for each emotion per date) supporting the conclusions of this article is available in the osf.io repository (https://osf.io/59qyk).

### Author contribution statement

Eric Mayor: Conceived and designed the experiments; Analyzed and interpreted the data; Contributed reagents, materials, analysis tools or data; Wrote the paper.

Marcel Miché: Conceived and designed the experiments; Wrote the paper.

Roselind Lieb: Conceived and designed the experiments; Wrote the paper.

### Funding statement

This research did not receive any specific grant from funding agencies in the public, commercial, or not-for-profit sectors.

### Declaration of interests statement

The authors declare no conflict of interest.

### Additional information

No additional information is available for this paper.
